# Social determinants and lifestyle factors for brain health: implications for risk reduction of cognitive decline and dementia

**DOI:** 10.1038/s41598-022-16771-6

**Published:** 2022-07-28

**Authors:** Susanne Röhr, Alexander Pabst, Ronny Baber, Christoph Engel, Heide Glaesmer, Andreas Hinz, Matthias L. Schroeter, A. Veronica Witte, Samira Zeynalova, Arno Villringer, Markus Löffler, Steffi G. Riedel-Heller

**Affiliations:** 1 Institute of Social Medicine, Occupational Health and Public Health (ISAP), Medical Faculty, University of Leipzig, Philipp-Rosenthal-Straße 55, 04103 Leipzig, Germany; 2 Global Brain Health Institute (GBHI), Trinity College Dublin, Dublin, Ireland; 3 Institute of Laboratory Medicine, Clinical Chemistry and Molecular Diagnostics, University Hospital Leipzig, Leipzig, Germany; 4 LIFE-Leipzig Research Center for Civilization Diseases, University of Leipzig, Leipzig, Germany; 5 Institute for Medical Informatics, Statistics and Epidemiology (IMISE), University of Leipzig, Leipzig, Germany; 6 Department of Medical Psychology and Medical Sociology, University of Leipzig, Leipzig, Germany; 7 Max Planck Institute for Human Cognitive and Brain Sciences, Leipzig, Germany; 8 Clinic for Cognitive Neurology, University Hospital Leipzig, Leipzig, Germany

**Keywords:** Psychology, Risk factors

## Abstract

Substantial evidence indicates a huge potential for risk reduction of cognitive decline and dementia based on modifiable health and lifestyle factors. To maximize the chances for risk reduction, it is useful to investigate associations of social determinants and lifestyle for brain health. We computed the “LIfestyle for BRAin health” (LIBRA) score for baseline participants of the Leipzig Research Centre for Civilization Diseases (LIFE) Adult Study, a population-based urban cohort in Germany. LIBRA predicts dementia in midlife and early late life populations, comprising 12 modifiable risk factors (heart disease, kidney disease, diabetes, obesity, hypertension, hypercholesterolemia, alcohol consumption, smoking, physical inactivity, diet, depression, cognitive inactivity). Associations of social determinants (living situation, marital status, social isolation, education, net equivalence income, occupational status, socioeconomic status/SES, employment) with LIBRA were inspected using age- and sex-adjusted multivariable linear regression analysis. *Z*-standardization and sampling weights were applied. Participants (n = 6203) were *M* = 57.4 (*SD* = 10.6, range 40–79) years old and without dementia, 53.0% were women. Except for marital status, all considered social determinants were significantly associated with LIBRA. Beta coefficients for the association with higher LIBRA scores were most pronounced for low SES (β = 0.80, 95% CI [0.72–0.88]; *p* < 0.001) and middle SES (β = 0.55, 95% CI [0.47–0.62]; *p* < 0.001). Social determinants, particularly socioeconomic factors, are associated with lifestyle for brain health, and should thus be addressed in risk reduction strategies for cognitive decline and dementia. A social-ecological public health perspective on risk reduction might be more effective and equitable than focusing on individual lifestyle behaviors alone.

## Introduction

Approximately 40% of all dementia cases could theoretically be prevented or at least delayed if certain risk factors were eliminated; including, among others, hypertension, obesity, diabetes mellitus, depression, physical inactivity and smoking^1^. The amounting epidemiological evidence on modifiable risk factors has led the World Health Organization (WHO) to issue recommendations on healthy lifestyle for risk reduction of cognitive decline and dementia^2^. With lack of curative treatment, risk reduction has become a key focus in global research and public health action plans to counteract the increasing number of people living with dementia due to population ageing^3^.

The multifactorial composition of dementia risk calls for risk reduction and preventive strategies which address its complexity. A multicomponent intervention approach was first successfully adopted by the Finnish Geriatric Intervention Study to Prevent Cognitive Impairment and Disability (FINGER)^4^. FINGER showed that a two-year lifestyle intervention that simultaneously targeted a range of modifiable risk factors (i.e. diet, physical exercise, cognitive training, vascular risk monitoring) led to maintained or improved cognitive function in older individuals at increased dementia risk^5^. A six-month multicomponent intervention improved cognitive function in older Chinese individuals with mild cognitive impairment (MCI)^6^. Other studies have been less conclusive^7–9^. However, many multicomponent trials in diverse populations are underway, and ongoing follow-up and long-term studies will show whether lifestyle interventions eventually prevent or delay dementia^10^. Evidence that dementia occurrence may indeed be modifiable comes from repeated cohort studies, which compare figures across birth cohorts: several meta-analyses concluded that dementia incidence is decreasing in Western high-income countries^11,12^. This is partially attributed to better education and management of cardiovascular disease; hence, improvement in modifiable risk factors^13^.

Now that risk reduction guidelines and lifestyle interventions are becoming increasingly important, it is crucial to find out who is at highest risk and may thus have a great need for intervention. Investigating social determinants in relation to modifiable health and lifestyle factors for brain health may help to achieve that. It is well established that social determinants, such as education, income, or employment status, influence health outcomes and explain systematic differences in health status^14^. Social determinants of health refer to the conditions in which individuals are born, grow, live, work and age^14^ and have been shown to impact cognitive function and dementia, particularly socioeconomic factors^15^. The “WHO conceptual framework for action on social determinants of health” argues that the effects of social determinants on health outcomes work through chains of mediating factors^16^. Lifestyle for brain health is likely a pathway of how social determinants affect cognition and dementia risk. It has long been argued and empirically underlined that lifestyle choices are heavily influenced by social determinants and constrained by social hierarchy^17,18^. Based on data of the English Longitudinal Study of the Aged (ELSA), Deckers et al. found that participants with lower wealth and lower education had a higher dementia risk and this was largely attributable to modifiable health and lifestyle risk factors^19^. Otherwise, studies on the relationship of social determinants and lifestyle specifically with regards to brain health are scarce.

### Study aims

The aim of the study was to investigate associations of social determinants and a validated index score of lifestyle for brain health in a midlife to early late life population without dementia. Results have implications for risk reduction strategies of cognitive decline and dementia.

## Methods

### Study design and recruitment

The Leipzig Research Centre for Civilization Diseases Adult Study (LIFE-Adult-Study) is a population-based cohort study with the purpose to investigate civilization diseases across the adult life span, conducted in Leipzig, an urban region in Germany. An age- and gender-stratified sample of individuals between 40 and 79 years of age was randomly drawn from population registers of the city of Leipzig. In addition, 400 more individuals between 18 and 39 years old were recruited from the same registry. The recruitment process was continued until an age- and sex-stratified sample size of n = 10,000 participants was reached. Exclusion criteria were pregnancy and insufficient German language skills. The response rate was 33%. Further study details have been previously described elsewhere^20^.

### Assessments

Assessments based on standard operation procedures (SOP) were conducted from August 2011 to November 2014 at the LIFE study center, University Hospital of Leipzig. SOP were carried out by trained study assistants under the supervision of experienced scientists. Assessments comprised computer-assisted personal interviews, for example for sociodemographic and socioeconomic variables, computer- or paper-based self-administered questionnaires, physical and medical examinations, psychometric tests, and clinical chemistry from blood and urine samples. The core assessment programme was absolved at an average duration of 5–6 h within one day. Assessments relevant for this study are detailed below.

### Social determinants of health

A structured computer-assisted interview on sociodemographic and socioeconomic variables provided information on relevant characteristics of the participants. Age and sex were already recorded as per recruitment. Social determinants of interest included the following sociodemographic variables: number of people living in a household (single and multi-person household), marital status (single; including widowed and divorced status and in partnership or married), and social isolation. Social isolation was assessed with the short form of the Lubben Social Network Scale (LSNS-6)^21^. The LSNS-6 measures social network in number and frequency of contacts with friends and family as well as social support received by them. It consists of six items, which are equally scored from 0 to 5 and adding up to a total score from 0 to 30. A score below 12 is considered an indicator of social isolation^21^. Social determinants with regards to socioeconomic characteristics comprised: education (low, middle, high; classified according to the Comparative Analysis of Social Mobility in Industrial Nations/CASMIN classification^22^), employment (employed, retired or unemployed), occupational status, and net equivalence income (NEI). Occupational status was operationalized as a household variable, i.e. if there lived more than one person in the household and there was a household member with a higher occupational status than the study participant’s status, the higher value of the respective household member was chosen. Assigned values ranged from 1 to 7, representing professional categories from (1) farmer/unskilled worker/semi-skilled worker to (7) freelance academics/civil servants in highest services/supervisors with ≥ 5 employees, based on the International Socio-Economic-Index of Occupational Status (ISEI) which uses the professional classification ISCO-88^23^. NEI refers to the needs-weighted net household income which was calculated according to the poverty and wealth reporting requirements of the German government^24^. It includes income from work, capital, transfers and other household members. Education, occupational status and NEI were used to operationalize the socioeconomic status (SES) according to a validated procedure described in Lampert et al.^25^ and classified into low, middle and high SES based on sample distribution.

### Lifestyle for brain health (LIBRA)

We computed the “LIfestyle for BRAin health” (LIBRA) score for all study participants aged 40–79 years^26^. LIBRA is a validated score that predicts cognitive decline and dementia in midlife and early late life populations, comprising 12 modifiable health and lifestyle factors: heart disease, kidney disease, diabetes, obesity, hypertension, hypercholesterolemia, alcohol consumption, smoking, physical inactivity, diet, depression, cognitive inactivity^27,28^. A standardized weight is assigned to each factor, reflecting its relative risk as extracted from systematic literature review and agreed upon in a Delphi consensus^29^. The weights are summed to yield the LIBRA score, ranging from − 5.9 to + 12.7. Higher scores indicate higher risk for cognitive decline and dementia, or “worse” lifestyle. In the LIFE-Adult-Study, we were able to compute the LIBRA score based on all 12 factors. The assessments, operationalization including cut-offs are detailed in Supplementary Table S1.

### Statistical analysis

Descriptive statistics were inspected with regards to sociodemographic and socioeconomic variables by calculating means with standard deviations and proportions. Furthermore, we computed the mean LIBRA score according to sociodemographic and socioeconomic strata, using t-tests or ANOVA, as appropriate. Multivariable linear regression analysis was performed to assess the association between social determinants and LIBRA, adjusted for age and sex. Model I included marital status, living situation, employment status and social isolation as well as education, occupational status, and NEI as separate indicator variables. Model II modified model I by including SES as an aggregated indicator and replacing the separate indicators of education, occupational status, and NEI. To allow for comparisons of the strength of associations of the social determinants with LIBRA, we *z*-standardized all independent variables. Results of the regression models are presented as standardized beta coefficients with corresponding 95% confidence intervals. Q–Q plots were used to evaluate the data distribution against the normal distribution for both models. A significance level of α = 0.05 (two-tailed) was applied. For all analyses, we adopted sampling weights to account for differences in sampling fractions in the LIFE-Adult-study compared to the general population. Specifically, the sampling weights were calculated based on the Leipzig 2012 population data from the Federal Statistical Office of Germany, using direct standardization in regard to the population stratum proportions of age and gender. Analyses were performed using the Stata 17.0 SE software package (StataCorp LLC).

### Ethics approval

The study protocol has been approved by the responsible institutional ethics board of the Medical Faculty of the University of Leipzig and adheres to the ethical standards laid down in the 1964 Declaration of Helsinki and its later amendments.

### Consent to participate

All persons gave their informed consent prior to their inclusion in the study.

## Results

### Sample characteristics

The LIBRA score was computed for 6203 out of 10,000 participants of the LIFE-Adult-Study. Five hundred thirteen (5.1%) participants were not eligible as they were younger than 40 years. Of the 9487 participants between 40 and 79 years of age, 3284 (34.6%) had missing values in at least one of the 12 variables needed for LIBRA. Excluded individuals with missing values tended to be older (*M* = 61.9 years, *SD* = 11.2; *t*(9,485) = 19.46, *p* < 0.001). The proportion of women did not differ (51.5%; *Χ*^2^(1) = 1.90, *p* = 0.168). The analytical sample had a mean age of 57.4 (*SD* = 10.6) years, with 53.0% women. The majority of participants had middle education (57.0%) and middle SES (60.3%). More sample characteristics are detailed in Table 1.

**Table 1:** Sample characteristics and mean LIBRA scores according to sociodemographic and socioeconomic strata in the LIFE-Adult-Study (n = 6203).

Variable	Sample N (%)	LIBRA score *M* (*SD*)	LIBRA Score diff *p* value
**Age, *M* (*SD*)**	57.36 (10.6)		
Midlife (40–59)	3536 (57.0)	− 0.06 (2.60)	0.847
Early late life (60–79)	2667 (43.0)	− 0.07 (2.60)	
**Sex**			
Women	3288 (53.0)	− 0.10 (2.59)	0.275
Men	2915 (47.0)	− 0.03 (2.61)	
**Education**			
Low	325 (5.3)	0.93 (2.41)	< 0.001
Middle	3511 (57.0)	0.43 (2.49)	
High	2329 (37.7)	− 0.96 (2.54)	
**Occupational status**			
1.0–1.9	59 (1.0)	1.06 (2.16)	< 0.001
2.0–2.9	877 (14.2)	1.08 (2.30)	
3.0–3.9	1627 (26.3)	0.45 (2.42)	
4.0–4.9	3229 (52.1)	− 0.51 (2.62)	
5.0–5.9	209 (3.4)	− 0.92 (2.54)	
6.0–7.0	192 (3.1)	− 1.53 (2.32)	
**Net household income, median (€)**	2600,00		
Below median	2327 (50.0)	0.21 (2.58)	< 0.001
Above median	2328 (50.0)	− 0.57 (2.50)	
**Socioeconomic status**			
Low	987 (15.9)	1.31 (2.29)	< 0.001
Middle	3742 (60.3)	− 0.01 (2.54)	
High	1474 (23.8)	− 1.12 (2.50)	
**Marital status**			
In partnership/married	3887 (62.7)	− 0.22 (2.57)	< 0.001
Single/divorced/widowed	2316 (37.3)	0.19 (2.63)	
**Living situation**			
Single household	1283 (20.7)	0.45 (2.62)	< 0.001
Multi-person household	4916 (79.3)	− 0.20 (2.58)	
**Employment**			
Employed	3680 (59.3)	− 0.21 (2.53)	< 0.001
Unemployed/housewife	423 (6.8)	1.10 (2.62)	
Retired	2098 (33.9)	− 0.05 (2.65)	
**Social isolation**			
Socially integrated	5199 (83.8)	− 0.21 (2.56)	< 0.001
Socially isolated	1004 (16.2)	0.71 (2.65)	

Annotations: Occupational status according to Lampert et al. ^25^, higher scores indicate higher status.

### LIBRA

LIBRA scores in the LIFE-Adult-Study ranged from − 5.9 to 9.1. Mean LIBRA scores did not differ between individuals in midlife vs. early late life (*M* = − 0.06, *SD* = 2.60 vs. *M* = − 0.07, *SD* = 2.60; *t*(6,201) = 0.19, *p* = 0.847) or between women and men (*M* = − 0.10, *SD* = 2.59 vs. *M* = − 0.03, *SD* = 2.61; *t*(6,201) = 1.09, *p* = 0.275). For all other social variables, LIBRA scores were significantly higher if the variable attribute indicated social disadvantage, for example, less education, less income or low SES, which is further detailed in Table 1.

### Social determinants of LIBRA

**Figure 1:**
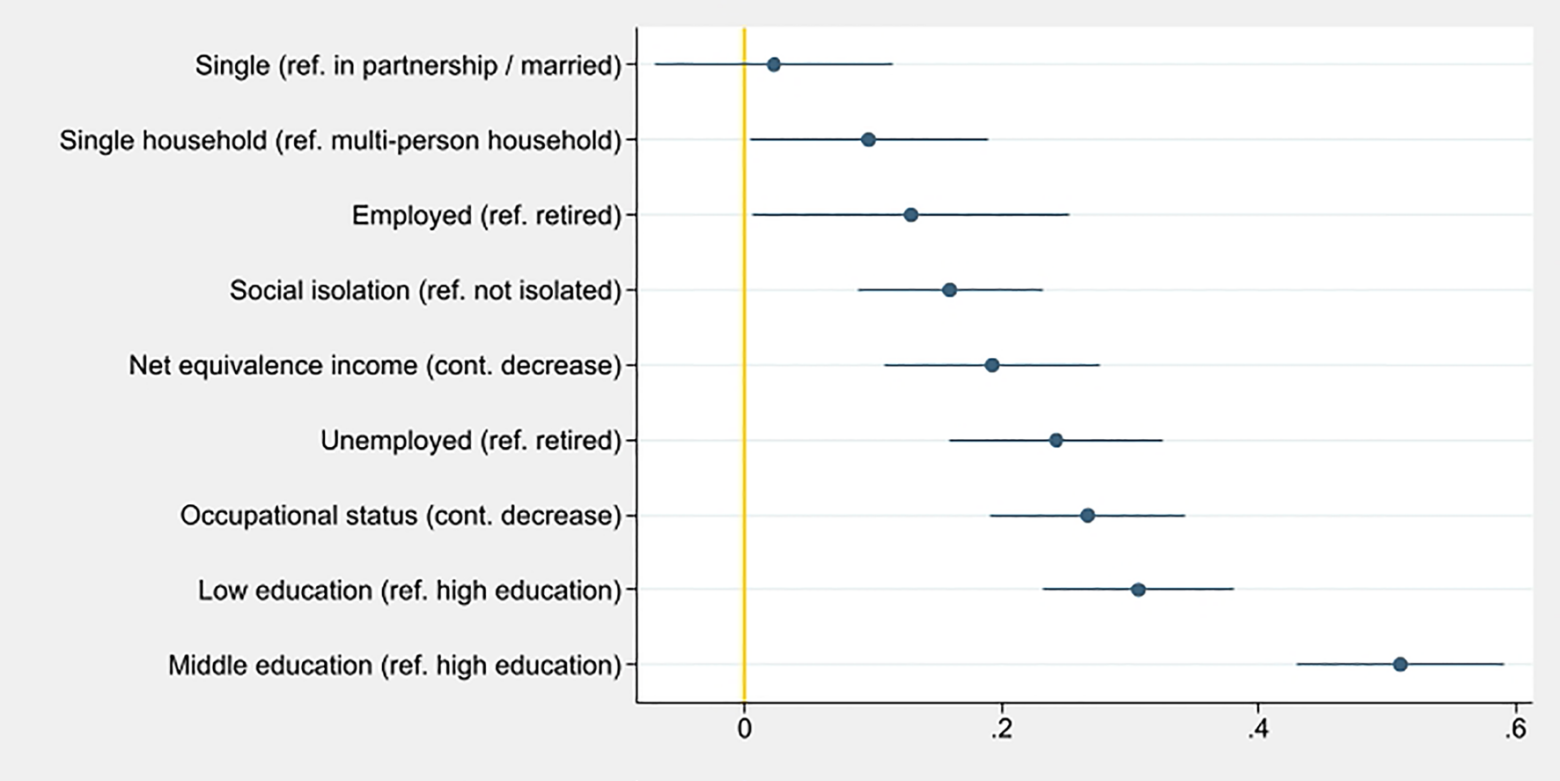
Beta coefficients and confidence intervals for associations of social determinants with lifestyle for brain health (LIBRA) scores, sorted according to strength of association of each factor for model I, in the LIFE-Adult-Study (n = 6203, age 40–79 years, without dementia).

Except for marital status, all considered social determinants were significantly associated with LIBRA in both models. In model I (Table 2, Fig. 1), beta coefficients for the association with higher LIBRA scores were highest for middle (β = 0.51, 95% CI [0.43–0.59], *p* < 0.001) and low education (β = 0.31, 95% CI [0.23–0.38], *p* < 0.001; Wald test for education: *Χ*^2^(2) = 168.84, *p* < 0.001), respectively; followed by lower occupational status (β = 0.27, 95% CI [0.19–0.28], *p* < 0.001) and unemployment (β = 0.24, 95% CI [0.16–0.33], *p* < 0.001; Wald test for employment: *Χ*^2^(2) = 33.82, *p* < 0.001).

**Table 2:** Results of the multivariable regression analysis (model I) on the association of social determinants and the lifestyle for brain health (LIBRA) score in the LIFE-Adult-Study.

Variable	Beta (95% CI)	SE	p
Age	0.12 (0.00–0.24)	0.06	0.047
**Sex**			
Women	Ref		
Men	0.08 (0.01–0.14)	0.03	0.024
**Education**			
High	Ref		
Middle	0.51 (0.43–0.59)	0.04	< 0.001
Low	0.31 (0.23–0.38)	0.04	< 0.001
Occupational status (cont. decrease)	0.27 (0.19–0.34)	0.04	< 0.001
NEI (cont. decrease)	0.19 (0.11–0.28)	0.04	< 0.001
**Marital status**			
In partnership/married	Ref		
Single/divorced/widowed	0.02 (− 0.07 to 0.11)	0.05	0.629
**Living situation**			
Multi-person household	Ref		
Single household	0.10 (0.00–0.19)	0.05	0.039
**Employment**			
Retired	Ref		
Employed	0.13 (0.01–0.25)	0.06	0.040
Unemployed	0.24 (0.16–0.33)	0.04	< 0.001
**Social isolation**			
Socially integrated	Ref		
Socially isolated	0.16 (0.09–0.23)	0.04	< 0.001

Annotations: Occupational status according to Lampert et al.^25^, higher scores indicate higher status.

**Figure 2:**
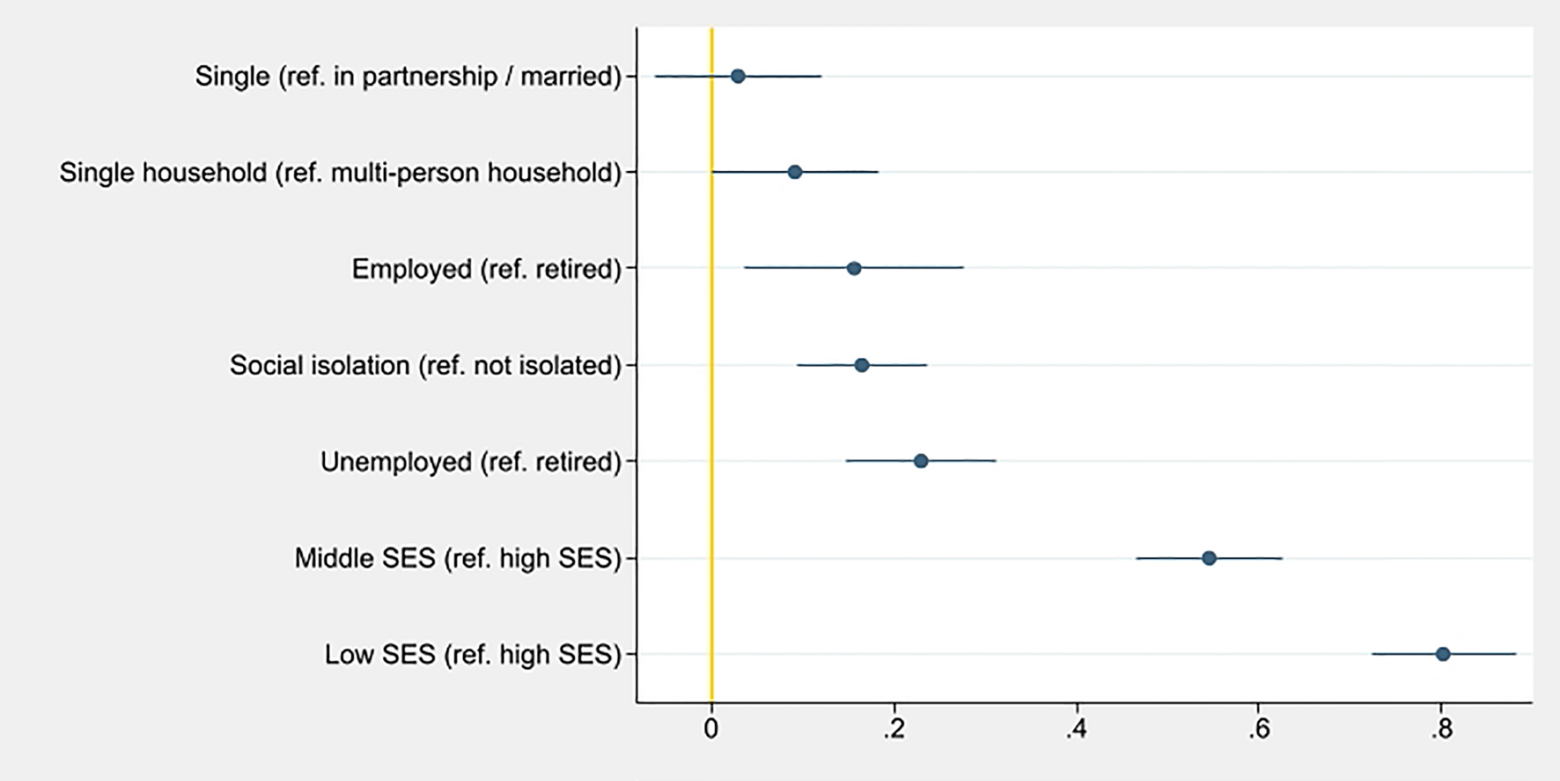
Beta coefficients and confidence intervals for associations of social determinants with lifestyle for brain health (LIBRA) scores, sorted according to strength of association of each factor for model II, in the LIFE-Adult-Study (n = 6203, age 40–79 years, without dementia).

In model II (Table 3, Fig. 2), beta coefficients for the association with higher LIBRA scores were highest for low SES (β = 0.80, 95% CI [0.72–0.88], *p* < 0.001) and middle SES (β = 0.55, 95% CI [0.47–0.62], *p* < 0.001; Wald test for SES: *Χ*^2^(2) = 402.83, *p* < 0.001), respectively, followed by unemployment (β = 0.23, 95% CI [0.15–0.32], *p* < 0.001; Wald test for employment: *Χ*^2^(2) = 29.71, *p* < 0.001). Overall, associations with factors indicating material circumstances were more pronounced than factors indicating social circumstances in both models. QQ-plots indicated approximately normally distributed data in both models.

**Table 3:** Results of the multivariable regression analysis (model II) on the association of social determinants and the lifestyle for brain health (LIBRA) score in the LIFE-Adult-Study.

Variable	Beta (95% CI)	SE	p
Age (increase)	0.11 (− 0.01 to 0.22)	0.06	0.072
**Sex**			
Women	Ref		
Men	0.05 (− 0.02 to 0.12)	0.03	0.132
**Socioeconomic status**			
High	Ref		
Middle	0.55 (0.47 to 0.62)	0.04	< 0.001
Low	0.80 (0.72 to 0.88)	0.04	< 0.001
**Marital status**			
In partnership/married	Ref		
Single/divorced/widowed	0.03 (− 0.06 to 0.12)	0.05	0.531
**Living situation**			
Multi-person household	Ref		
Single household	0.09 (− 0.00 to 0.18)	0.05	0.050
**Employment**			
Retired	Ref		
Employed	0.16 (0.04 to 0.28)	0.06	0.010
Unemployed	0.23 (0.15 to 0.32)	0.04	< 0.001
**Social isolation**			
Socially integrated	Ref		
Socially isolated	0.16 (0.09 to 0.24)	0.04	< 0.001

## Discussion

We aimed to investigate associations of social determinants and lifestyle factors for brain health in a midlife to early late life population without dementia. Quantifying dementia risk using the lifestyle for brain health (LIBRA) index score, which comprises 12 weighted modifiable health and lifestyle risk factors, we found that all, but one social determinant (i.e. marital status) under investigation were associated with LIBRA: less education, lower occupational status, lower net equivalence income (NEI)—when summarized as socioeconomic status (SES), lower SES—as well as social isolation, living alone and being unemployed and being employed in reference to being retired were associated with higher LIBRA scores, thus poorer lifestyle known to be associated with increased risk for cognitive decline and dementia. Overall, the associations were most marked for SES and education, occupational status as well as unemployment, potentially indicating that material circumstances may have a larger effect on lifestyle for brain health than social circumstances, such as number of people living in a household or social network. Moreover, the more pronounced association of SES with LIBRA, combining education, occupational status and NEI, compared to the individual associations of these three factors, suggests that disadvantages in social determinants cluster and could cumulate in more adverse lifestyle. This is consistent with Cockerham's healthy lifestyle theory, which posits that lifestyle is not random individual behaviour but a pattern of aggregated routines based on choices from a range of options dependent on social status^17^. Thus, social determinants may channel lifestyle choices, with less options for socially disadvantaged individuals.

The significant associations of social determinants, particularly socioeconomic factors, and lifestyle for brain health may hint towards a relevant pathway for risk for cognitive decline and dementia. As Deckers et al. suggested, lifestyle for brain health explains a large proportion of dementia risk in individuals with high vs. low wealth^19^. Low SES can constitute a barrier for healthy lifestyle with regards to affordability of foods, time constraints due to long work hours with little pay, or poor housing^30,31^. A German study found that minimum wage and financial support for long-term unemployed individuals were insufficient to assure a healthy lifestyle^32^. In our study, lower household income and unemployment were associated with poorer lifestyle for brain health compared to higher income or being employed or retired. Retired participants had lower lifestyle risk scores than employed individuals. It has been argued that transitioning into retirement could lead to less time constraints and stress associated with employment; thus opening up resources for more healthy behaviours, for example, more sleep and leisure activities^33,34^. Notably, this was independent of age. Age was not associated with differential lifestyle which is in line with previous studies which suggested that social class-related health lifestyles become 'locked in' with increasing age unless emerging age-related chronic diseases, for example diabetes, would motivate changing lifestyle behaviours^35,36^.

While less education is considered a risk factor for dementia^1^, not all studies reported a direct association, and how education and dementia are eventually linked remains under debate^37–39^. As opposed to low education, for example, individuals with high educational attainment may have a greater brain reserve to tolerate pathological changes in brain structure and greater cognitive reserve that can delay the clinical manifestation of dementia^40^. Moreover, higher levels of education may be associated with healthier lifestyle, as found in our study, and in line with studies that suggested education is rather indirectly related with dementia risk by wealth and lifestyle differences^19^. Contrary, Ngandu et al.^37^ suggested the association between low education and dementia might not be explained by unhealthy lifestyles of the less educated but rather by greater cognitive reserve in higher educated individuals. Furthermore, cross-country-results have indicated that the effect of education on cognitive function may offset the adverse implications of living with low income^41^. A disentangling of the seemingly complex relationship of education, other socioeconomic factors, lifestyle for brain health and subsequent cognitive decline and dementia is desirable as it has implications for risk reduction strategies.

In addition to social determinants, which point to socioeconomic aspects and thus dictate material circumstances, social aspects were also linked to lifestyle for brain health, but to a lesser extent. Social isolation and living alone in a household were both associated with poorer lifestyle for brain health, independent of socioeconomic factors, which is in line with previous findings investigating general lifestyle behaviours^42^. Moreover, findings suggest an interrelation of material and social circumstances, for example, social support is lower in unemployed individuals and those with financial difficulties^43^. Living in a single household is associated with higher costs of living, again pointing to a clustering of social disadvantage^44^. While studies suggested that marital status is associated with dementia in that married individuals are at lower risk, the findings of our regression analysis did not suggest that such a linked is related to lifestyle factors^45–47^.

### Strength and limitations

Strength of the study include a large sample of individuals without dementia in midlife to early late life, which is thought to be the age range most relevant for risk reduction. Moreover, we were able to operationalize the LIBRA score with all 12 factors, thus being able to utilize its full spectrum. However, due to data availability in our study and country-specific guidelines, operationalization of some factors (e.g. diet, alcohol consumption, high cognitive activity) may differ to other studies using the LIBRA score, compromising comparability of results between studies. Another limitation is that a third of the study participants had to be excluded from analysis due to missing values. Excluded individuals were older than included individuals. It is likely that reported associations are thus an underestimation. However, when repeating all analyses with an abbreviated LIBRA score (excluding the factor “high cognitive activity”, which had the highest number of missing values), results did not differ. Generalizability of findings may further be compromised by a participation rate of 33% in the LIFE-Adult-Study, which reflects an observed trend of declining willingness to participate in epidemiological studies^48^. Moreover, despite drawing on theory of how social disadvantage leads to poor lifestyle, this study uses cross-sectional observations, which does not allow for valid conclusions on the temporality of associations, and should therefore be confirmed by longitudinal studies.

We were only able to consider a limited number of social determinants due to data availability in our study. There are many other factors shaping the complex conditions of an individual’s life and lifestyle. Particularly, our factors were limited to individual-level social determinants, whereas characteristics of the environment (for example, the neighbourhood, built environment and health care infrastructure) are of relevance as well and should be subject to future studies. Furthermore, the LIBRA score contains protective and risk factors which are weighted against each other, leading to a general interpretation of “better” or “worse” lifestyle; however, the twelve factors in LIBRA cover a big set of health and lifestyle factors, which are interrelated in complex ways. Overall, LIBRA cannot address any of the complexity in the interplay between factors.

### Implications for risk reduction of cognitive decline and dementia

Social determinants should be addressed in risk reduction of cognitive decline and dementia. Current risk reduction strategies are heavily focused on individual-level lifestyle interventions^2^. While such interventions are important and have shown benefits for older people at increased risk of dementia^5^, we must recognize that the scope of lifestyle interventions may be limited if contextual factors are not considered. In addition, recruitment of socially disadvantaged older individuals remains a challenge due to various structural barriers, leading to underrepresentation in health trials^49,50^. This calls for a diversified focus in public health action.

Risk reduction strategies for cognitive decline and dementia are at risk for the so called "lifestyle drift". This refers to the tendency in public health to focus on individual behaviours, but ignoring the drivers of these behaviours^51^. This is problematic as this approach shifts the responsibility for unhealthy behaviour largely onto the individual, which is unfair against the background of social inequity. As studies like ours point to the relevance of social determinants in lifestyle for brain health, adopting a social-ecological model, i.e. recognizing environmental factors, individual factors and their interactions, in risk reduction of cognitive decline and dementia could, first, help to better exploit the huge potential of risk modification, and, second, help to reduce inequities in dementia risk^52,53^. Specifically, under the assumption that conditions shape lifestyles, a top policy priority should be enabling good basic living standards for everyone. This requires pro-social legislations in favour of a sustainable needs-based economy, which allows for, for example, universal public education, equal health care access, legal minimum wages as well as fair income distribution and taxation.

## Conclusion

Social determinants are linked with lifestyle for brain health and should therefore be addressed in efforts aiming at reducing the risk of cognitive decline and dementia. Robust evidence on the relationship between social determinants and lifestyle factors for brain health is pivotal to inform policy and call politicians to action to move towards effective and equitable risk reduction of cognitive decline and dementia.

## Supplementary Information

Supplementary Information.

## Data Availability

Data used in this study can be made available to researchers on request to the correspondent author.
