# Supplementary material for: Social determinants and lifestyle factors for brain health: implications for risk reduction of cognitive decline and dementia
Source: Sci Rep. 2022 Jul 28;12:12965. doi: 10.1038/s41598-022-16771-6 (PMC9334303; doi:10.1038/s41598-022-16771-6)
Supplement: Supplementary file 1 — Supplementary Information. [file 41598_2022_16771_MOESM1_ESM.pdf]

Table S1. Operationalization of the Lifestyle for Brain Health (LIBRA) score and factor weights in the Leipzig Research Centre for Civilization Diseases Adult Study (LIFE).				
**Factor**	**Assessment**	**Operationalization and cutoffs**	**N (missings)**	**Weight**
Heart disease	Self-reported medical history	Any of: myocardial infarction, angina pectoris, arrhythmia, cardiac insufficiency	9786 (214)	+1.0
Diabetes mellitus	Oral glucose tolerance test (oGTT) was conducted in all fasted participants who came to the study centre before 8 a.m. After initial blood drawing and an intake of 75 g of glucose solution (Accu-Chek® Dextrose O.G.T. Saft, Roche Diagnostics Deutschland GmbH), blood samples were taken for measurement of glucose and insulin concentration at 30 (glucose only) and 120 min; self-reported medical history	According to established cutoffs of the World Health Organization (WHO) guidelines [1]: hemoglobin A1c (HbA1c) ≥ 6.5% (≥ 48 mmol/mol); if HbA1c was not available: fasting glucose ≥ 7.0 mmol/l; oral glucose tolerance test (oGTT; 120 min) ≥ 11.0 mmol/l; self-reported diabetes	10000 (0)	+1.3
Hypercholesterolemia	Laboratory test performed on fresh biospecimen directly on the day of sample collection; including total cholesterol, high-density lipoprotein (HDL), low-density lipoprotein (LDL) cholesterol	According to the guidelines of the German Cardiac Society[2]: raw value of total cholesterol of >= 5.2 mmol/l and low-density lipoprotein (LDL) of >= 4.2 mmol/l	9772 (228)	+1.4
Hypertension	Blood pressure was measured three times at 3-min intervals using an automatic oscillometric blood pressure monitor (OMRON 705IT, OMRON Medizintechnik Handelsgesellschaft mbH) after resting for at least 5 min at the study center; self-reported medical history	According to WHO guidelines [3]: mean systolic blood pressure ≥ 140 mm H;, or, if not available, diastolic blood pressure ≥ 90 mm Hg, or self-reported hypertension	9987 (13)	+1.6
Depression	20-item version of the Centre of Epidemiological Studies Depression Scale (CES-D)[4]; self-reported medical history	CES-D score ≥ 16 or, if not available, self-reported depression	9431 (569)	+2.1
Obesity	Body mass index (BMI) based on weight and height measurement. Body weight was measured with an electronic scale (SECA 701, Seca Gmbh & Co KG) with a precision of 0.01 kg. Body height was assessed by means of a stadiometer (SECA 240) to the nearest 0.1 cm	Established cut-offs according to WHO guidelines [5]: BI) ≥ 30; if BMI was not available: waist circumference for women > 88 cm, for men > 102 cm	9980 (20)	+1.6

	Waist circumferences; taken using an ergonomic circumference measuring tape (SECA 201) to the nearest 0.1 cm			
Smoking	Standardized self-report questionnaire on ever and current smoking.	Self-reported current smoker	9590 (410)	+1.5
Low-to-moderate alcohol consumption	Standardized self-report questionnaire on frequency and amount of different alcoholic beverages consumed within the last year	Estimate of consumed grams of alcohol per day; low-to-moderate drinking as any amount of up to 12g/day for women, 24g/day for men according to German guidelines [6]	9756 (244)	-1.0
Physical inactivity	Subjectively reported physical activity was assessed using the short form of the International Physical Activity Questionnaire (IPAQ-SF)[7]	Total metabolic equivalent of task (MET) according to IPAQ-SF; "low" [= physically inactive], i.e. less than moderate level nominally indicating meeting physical activity guidelines of 30 minutes of moderate intensity activity 5 days a week, 20 minutes of vigorous activity 3 days a week, or a combination	8270 (1730)	+1.1
High cognitive activity	Indices of occupational cognitive demands were based on data from the 2010 Standard Occupational Classification of the O*NET database (http://www.onetonline.org)[8] by the US Department of Labor/Employment and Training Administration (USDOL/ETA). O*NET contains standardized validated descriptors for a wide range of occupations, including associated level and importance of cognitive abilities. We first translated the main occupation of each LIFE-Adult Study participants into English, and then matched the corresponding O*NET code to the occupation. O*NET data on cognitive abilities comprised 7 descriptors (e.g., memorization, deductive reasoning, verbal abilities) with a rating of the degree of its importance (1 = not important, 5 =	High cognitive activity being assumed for those in the highest tertile	7900 (2100)	-3.2

	extremely important) and the level of how much a descriptor is needed to perform an occupation (0 = not at all, 7 = very much). The average of descriptors for importance and level was calculated, and both factors were calculate to achieve a continuous variable of overall occupational cognitive demand. The score was then split into tertiles.Thanks			
Healthy diet	Regarding dietary patterns, participants were asked about the consumption frequency of 34 food groups and 13 types of beverages within the last 12 months	Self-report of intake of several portions of fruit and/or vegetable daily over the last 12 months as assessed using a food frequency questionnaire	9779 (221)	-1.7
Chronic kidney disease	Laboratory test performed on fresh biospecimen directly on the day of sample collection; serum creatinine blood test;<br>self-reported medical history	Estimated glomerular filtration rate (eGFR) calculated using the cystatin C-based chronic kidney disease (CKD) epidemiology collaboration equation[9], eGFR cutoff < 60 mL/min/1.73m^2; or, if not available, self-report of kidney insufficiency, any other kidney disease, dialysis duty	9401 (599)	+1.1

References

1. World Health Organization. *Classification of diabetes mellitus*. Geneva: World Health Organization; 2019. https://apps.who.int/iris/handle/10665/325182.
2. Leitlinien der Deutschen Gesellschaft für Kardiologie – Herz- und Kreislaufforschung e.V. https://leitlinien.dgk.org/. Updated January 14, 2022. Accessed January 14, 2022.
3. 1999 World Health Organization-International Society of Hypertension Guidelines for the Management of Hypertension. Guidelines Subcommittee. *J Hypertens*. 1999;17(2):151-183.
4. Radloff LS. The CES-D Scale. *Applied Psychological Measurement*. 1977;1(3):385-401. doi:10.1177/014662167700100306.
5. World Health Organization. *Waist circumference and waist-hip ratio : report of a WHO expert consultation, Geneva, 8-11 December 2008*. Geneva: World Health Organization. 2011. https://apps.who.int/iris/handle/10665/44583.
6. Basisinfo_Alkohol. https://www.dhs.de/fileadmin/user_upload/pdf/Broschueren/Basisinfo_Alkohol.pdf. Accessed January 14, 2022.

7. Hagströmer M, Oja P, Sjöström M. The International Physical Activity Questionnaire (IPAQ): a study of concurrent and construct validity. *Public Health Nutr*. 2006;9(6):755-762. doi:10.1079/phn2005898.
8. Handel MJ. The O*NET content model: strengths and limitations. *Journal for Labour Market Research*. 2016;49(2):157-176. doi:10.1007/s12651-016-0199-8.
9. Inker LA, Schmid CH, Tighiouart H, et al. Estimating Glomerular Filtration Rate from Serum Creatinine and Cystatin C. *New England Journal of Medicine*. 2012;367(1):20-29. doi:10.1056/NEJMoa1114248.
